# Poisoning by Matches

**Published:** 1875-07

**Authors:** 


POISONING BY MATCHES.—Phosphorus, largely used in making friction matches, is a powerful poison when swallowed, inducing bloody discharges from the bowels and bladder, and great derangement of the nerve-centres. If swallowed, the antidote is spirits of turpentine, diluted with milk. An adult should take an ounce of turpentine, and if this is vomited, more should be given. If retained, a quarter of an ounce the next day, will usually complete the cure. It is believed that phosphorus loses its power to give off toxic vapors and other influences destructive to life, in the presence of turpentine.

---

DISGUISING UNPLEASANT MEDICINE.—If a person thinks he may be made wiser, or better, or happier, by swallowing some castor oil, he will naturally desire to make the dose as palatable as possible. The unpleasant flavor of this oil, as well as cod-liver oil, and many other medicines, may be concealed by squeezing half an orange into a glass, and pouring the oil upon the expressed juice. All disturbance of the liquids must then be avoided, and the juice from the other half of the orange must be squeezed over the oil. Thus enclosed between the two palatable layers, the oil may be swallowed without perception of its flavor.

---

SUBSTANCES IN THE EAR.—If a pea or other hard substance gets into the ear, it will be quite useless to endeavor to remove it by passing an instrument over it, and pressing downwards, as the form of the channel is such that this act will simply force it back upon the membrana tympani. A strong light should be thrown into the ear by reflection, and a delicate instrument, like a dentist's excavator must be pushed carefully *under* the obstruction, when the point, curved at a right angle, may be gently forced into it, and it may be withdrawn with ease.

---

THE "SHINGLES.—The peculiar form of Herpes, popularly called "Shingles," is an eruption corresponding to the termination of certain cutaneous nerves. Applications to the surface prove of very slight avail in this annoying disease. The affection is really an interference with the function of absorption. The severe pain, the almost unendurable itching, and the great inflammation, are all quickly relieved by subcutaneous injections of morphia.

